# Prevalence and Molecular Diversity of Plant-Parasitic Nematodes of Yam (*Dioscorea* spp.) in China, with Focus on *Merlinius* spp.

**DOI:** 10.3390/biology10121299

**Published:** 2021-12-09

**Authors:** Sulaiman Abdulsalam, Huan Peng, Yingjuan Yao, Linjuan Fan, Ru Jiang, Hudie Shao, Yingdong Zhang, Wenkun Huang, Ling’an Kong, Deliang Peng

**Affiliations:** 1 State Key Laboratory for Biology of Plant Diseases and Insect Pests, Institute of Plant Protection, Chinese Academy of Agricultural Sciences, Beijing 100193, China; asulaiman@abu.edu.ng (S.A.); penghuan@caas.cn (H.P.); jiangruby@126.com (R.J.); shaohudie@126.com (H.S.); zhangyingdong26@163.com (Y.Z.); wkhuang@ippcaas.cn (W.H.); konglingan@caas.cn (L.K.); 2 Department of Crop Protection, Division of Agricultural Colleges, Ahmadu Bello University, Zaria 810107, Nigeria; 3 Institute of Applied Agricultural Micro-Organisms, Jiangxi Academy of Agricultural Sciences, Nanchang 330200, China; yaoyingjuan@webmail.hzau.edu.cn (Y.Y.); fljx99@163.com (L.F.)

**Keywords:** yam, *Pratylenchus coffeae*, *Meloidogyne* spp., *Merlinius brevidens*, morphology, molecular

## Abstract

**Simple Summary:**

Plant nematodes (PPNs) have been documented as economically important pests of yam in different parts of the world with *Pratylenchus* spp. and *Meloidogyne* spp. being the most widespread and destructive pests in Asia, causing significant yield losses. The primary inspiration for this study is the scarcity of information about nematode pests of yam in China and the fact that no previous study has looked into the prevalence, diversity, and integrative taxonomy of PPNs species in China cropping systems, as well as their consequences on yam cultivation. Therefore, the objectives of this study were to conduct a nematode study in south-eastern China to (i) record the prevalence and diversity of PPNs of cultivated yam with emphasis on the five prominent genera identified, and (ii) characterized morphologically, morphometrically, and molecularly *Merlinius* spp. The high prevalence of the *Merlinius* spp. revealed that the species may be more important to yam than previously thought. Conclusively, this study provided useful baseline PPNs data for yam in China. Hence, future study should focus on; developing innovative, environmentally friendly, and cost-effective nematode management strategies to control this pest that damage farmers’ yam fields in China, and constantly updating information of PPNs species found to allow proactive measures in terms of their spread and management.

**Abstract:**

There is little information about nematode pests associated with yam in China. Between 2020 and 2021, surveys of yam fields were conducted to investigate the abundance and prevalence of plant-parasitic nematodes in major yam growing areas. A total of 110 bulk soil samples from the yam rhizosphere and 48 yam tubers were collected from seven counties in Jiangxi and Shandong provinces. Standard protocols were used to extract nematodes from soil and tubers and identified at the genus level. In this study, 16 species and 13 nematode genera were recorded. The five most prominent species on the yam rhizosphere according to mean population densities were *Pratylenchus coffeae* (291/individuals), *Meloidogyne* (262/individuals), *Rotylenchulus reniformis* (225/individuals), *Merlinius* (224/individuals), and *Helicotylenchus dihystera* (171/individuals). In the tubers, the three most prominent species were *Pratylenchus coffeae* (415/individuals), *Meloidogyne* (331/individuals), and *Rotylenchulus reniformis* (115/individuals). These species were verified with appropriate molecular analysis. The high prevalence of the ectoparasite (*Merlinius* spp.) on the rhizosphere of yam also revealed that *Merlinius* spp. May be more important to yam than previously thought. Morphological and molecular analyses further confirmed the identity of the species as *Merlinius brevidens* and were characterized for the first time on yam in China. Minor morphometrical differences (slightly longer body and stylet) were observed in Chinese populations of *M. brevidens* compared to the original description. Additionally, this study reveals that *M. brevidens* isolated from China showed a higher nucleotide sequence in the ITS region compared to *M. brevidens* populations from India. This finding provides baseline information on the nematode pest occurrence on yam in China and calls for effective management.

## 1. Introduction

Yam belongs to the genus *Dioscorea* (family; Dioscoreaceae) consisting of about 644 species with underground tubers [1]. The three most common cultivated species include *D*. *rotundata*, *D*. *alata*, and *D*. *cayenensis* [2], while *D*. *esculenta*, *D*. *nummularia*, *D*. *bulbifera*, *D*. *pentaphylla*, *D*. *transversa*, *D*. *japonica*, *D*. *trifida*, *D*. *dumetorum*, and *D*. *opposita* are also of economic importance. *Dioscorea opposita* is the only species cultivated in temperate areas and is commonly grown in China and Japan [3]. Yam is an important staple food crop in tropical and subtropical areas of Africa, Asia, South America, the Pacific, and the Caribbean region [4,5]. It is the fourth most important tuber crop after potato, cassava, and sweet potato globally, with West Africa accounting for about 92% (67 MT) of the total output annually [6]. Though China’s yam output was not included in the FAO statistics, it is currently a significant and isolated center of yam domestication [7]. In China, approximately 5 to 6 MT of yam are produced every year through the planting of diverse species in most yam-producing areas such as Jiangsu and Shandong provinces [7,8].

In China, consumption of yam is on the increase due to the recent belief that, nutritionally, its reserves of micronutrients, minerals, and proteins can be used to address potential deficiencies [7,9]. Additionally, its production brings in income to the country [5,7]. However, yam is susceptible to numerous diseases, particularly bacterial dry rot (*Corynebacterium* spp.), anthracnose (*Colletotrichum* spp.), viruses, and nematodes [4,10]. It is highly susceptible to plant-parasitic nematodes (PPN) especially the yam nematode (*Scutellonema bradys*), root-lesion nematodes (RLN; *Pratylenchus* spp.), and root-knot nematodes (RKN; *Meloidogyne* spp.). Plant-parasitic nematodes interacts with other plant pathogens, resulting in increased damage caused by other diseases and affecting global food supplies [4,5]. Yam infected with RKN has been reported to show galls and “crazy root” syndrome on tubers, altering tuber appearance and reducing tuber quality. *Pratylenchus* spp. and *S*. *bradys* also contribute to dry rot disease and tuber surface cracking, which in turn influence the production and quality of tuber [4,11,12]. Additionally, tubers with nematode symptoms are less appealing and so have poor market value [4,11,12]. The association of yam to PPN has been reported in China with RKN responsible for the loss of several cultivars [4,5]. About 24–80% yield reduction in yam in China is associated with RKN (*M*. *arenaria*), while about 30–100% is attributed to RLN (*P*. *coffeae*) [4].

Stunt nematodes of the genus *Merlinius* Siddiqi, 1970 include 34 nominal species [13]. These nematodes are migratory ectoparasites of a wide range of plants globally and are ubiquitous in agricultural settings. The accurate identification of these stunt nematodes from both rhizosphere soil and root samples is critical for successful disease management [13,14,15]. The majority of species attack and feed on plant roots and underground plant parts, resulting in low to moderate yield losses in several crops, including rice (*Oryza sativa* L.), wheat (*Triticum* spp.), maize (*Zea mays*), and potato (*Solanum tuberosum* L.) [13,15]. Allen [14] first described a species of this genus, *Merlinius brevidens*, as a major pathogen of grasses in the USA and several other countries [13,15]. Although this species is not among the species considered most important to yam (*Dioscorea* spp.), some of them can become destruction agents when populations exceed economic threshold levels. [13,16]. They are mostly recognized for the damage they caused to grasses and cereals through feeding on epidermal cells, resulting in root and foliage stunting, leaf yellowing, defoliation, and wilting [16]. Morphological identification of *Merlinius brevidens* is characterized by the absence of areolated lateral fields; six longitudinal incisures; female tail sub-cylindrical (c’ = 2.4–4.6) with rounded terminus; and robust stylet [17,18]. Males are rarely seen and unlikely to be required for reproduction. However, the substantial intraspecific diversity of key diagnostic features makes morphological identification of this genus challenging, and its evolutionary relationships with other genera in the same family are complex, making it problematic and contentious [16,17,18]. To elucidate the identity and evolutionary relationships of the species in this genus and related genera, morphological and molecular analyses are required [13,17,18].

Plant-parasitic nematodes infecting yam must be identified accurately and promptly to develop appropriate management methods that will reduce losses. This is particularly important for yam due to the diversity of nematode species found in different yam-growing regions across the world [4,11,19]. Accurate nematode identification enables the differentiation of regulated and non-controlled nematode pests, as well as the exclusion of species under quarantine or regulatory measures. The prevalence and geographical distribution of PPNs in Asia were reviewed by Bridge et al. [11] who reported that eight species (*P*. *coffeae*, *M*. *incognita*, *M*. *arenaria*, *M*. *hapla*, *Radopholus similis*, *Paratrichodorus porosus*, *Rotylenchulus reniformis*, and *Helicotylenchus dihystera*) were found damaging yam. In China, only three species of PPNs (*P*. *coffeae*, *M*. *incognita*, and *M*. *arenaria*) have been reported in association with yam [4]. However, many other species were not reported and verified with appropriate molecular analysis. The current availability of molecular methods may aid in the identification of nematodes by providing tools for species differentiation. Therefore, ribosomal DNA sequences from partial 18S, ITS regions, and the D2-D3 expansion segments of the 28S, as well as mitochondrial DNA (mtDNA) sequences, have proven to be useful diagnostic tools for the characterization and establishment of phylogenetic relationships within PPNs, particularly when morphological features may cause doubt in interpretation [14,15]. Integrative taxonomy, which combines molecular methods with morphology and morphometry methods to diagnose the identified species, is critical for accurate nematode identification. As a result, in this study we conducted a comprehensive nematode survey in the two main provinces of China yam production areas, with the following objectives: (a) to determine the prevalence of PPNs infesting yam in southeastern China; (b) to characterize *Merlinius* species using integrative taxonomy; (c) to study the phylogenetic relationships of the identified *Merlinius* species with other related species.

## 2. Materials and Methods

### 2.1. Extraction, Identification, and Morphological Characterization of Nematodes

Soil and tuber samples were collected from Jiangxi and Shandong provinces of China in April 2020 and January 2021 (Table 1). A total of 110 bulk samples were collected on the rhizosphere of different yam crops at 5 to 10 cm from the yam vine base and each bulk sample comprised four subsamples taken from four different plants of the same cultivar in the fields. Along with the four diagonal points of each selected plant, soil samples around the roots, about 25–30 cm depth, were gently dug out using a hoe to cover the rhizosphere as much as possible. The 48 tubers, together with soil samples, were put in a polyethylene bag, sealed, and labeled accordingly before being transported in cool insulated bags to the Nematology Laboratory of Institute of Plant Protection, Beijing, and stored at 4 °C in the cold room before nematodes were extracted within one week of collection. Nematodes were recovered from yam rhizosphere and tubers using the modified Baermann tray technique and Whitehead tray immersion technique [20,21]. Extraction from the rhizosphere was established using 100 mL soil sub-samples per sample, which included all roots recovered from the soil. Tubers were peeled, chopped with a kitchen knife, and three sub-samples of 5 g tuber peels were used for nematode extraction [12].

The data obtained were subjected to community analysis to determine the frequency of occurrence (FO) of each genus and the mean population density (MPD) according to Bello et al. [22]:

FO = (number of samples containing a nematode species ÷ number of samples collected) × 100

Mean population density (MPD) = total number of nematodes ÷ number of samples obtained across the nine locations sampled and also for pooled data of the two provinces

#### 2.1.1. Morphological Characterization

The five prominent species were individually picked, killed by gentle heat, and mounted on temporary glass slides with cover glass supported by a wax ring, and some were fixed with formaldehyde: glycerin (4:1). Morphological measurements were carried out using the Cells Sens Standard program and Photographed using an Olympus, DP80 digital camera attached to an Olympus BX53 microscope (Olympus Corporation Shinjuku Monolith, 2-3-1 Nishi-Shinjuku-Ku, Japan).

#### 2.1.2. Molecular Characterization

The genomic DNA extraction method was similar to those described by Htay et al. [23]. The rDNA-ITS and D2-D3 target genes of the nematode populations were amplified by PCR (Bio RAD, S1000™ Thermal Cycler). The PCR reaction was 20 μL comprising of 0.5 μL each of the universal primers; rDNA1 (5′-ACGAGCCGAGTGATCCACCG-3′) and rDNA2 (5′-TTGATTACGTTCCCTGCCCTTT-3′) for the ITS region [23]; D2A (5′-ACAAGTACCGTGAGGGAAAGTTG-3′) and D3B (5′-TCGGAAGGAACCAGCTACTA-3′) for the D2-D3 region [24]; 2 μL template DNA; 10 μL 10× PCR buffer (with Mg^2+^), (Takara Company, Beijing, China); and 7 μL sterile ddH_2_O. PCR amplification was carried out in a thermal cycler S1000 with the following conditions; 94 °C for 4 min; 35 cycles at 94 °C for 45 s; 50 °C for 30 s; 72 °C for 1 min; and 72 °C for 10 min (ITS), and 94 °C for 4 min; 35 cycles at 94 °C for 1 min; 55 °C for 1 min; 72 °C for 2 min; and 72 °C for 10 min (D2-D3). After the PCR, the amplified DNA products were run on a 1.0% agarose gel electrophoresis and visualized on a trans-illuminator (UV light) and photographed (BIO-RAD-Gray scale Digital Camera, CFW, Hercules, CA, USA). The amplified PCR products were purified using TIAN-gel Midi Purification Kit (TIANGEN). The purified PCR products (DNA) were ligated to the p^MD19-T^ Vector and transformed into DH5 alpha Competent Cells. The PCR amplification was further confirmed with the insertion of the primer and the band expected. The DNA fragments were sent to the BGI Biotechnology Company, Limited, Beijing, China for sequencing.

For phylogenetic analysis, the generated sequences (28S-rRNA and ITS-rRNA) of the studied stunt nematode populations were aligned using the Clustal X 1.83 software [25], with previous sequences of related species deposited in the GenBank database. Sequences alignments were analyzed with Bayesian inference (BI) using MrBayes 3.2.6 [26] and for each dataset, outgroup taxa were chosen based on previous study [13]. The general time-reversible substitution model with invariant site estimation and four-category gamma distribution (GTR + I + G) was selected as the suitable nucleotide substitution model used in the two studies (28S-rRNA and ITS-rRNA). For each gene, BI analysis was initiated with a random starting tree and was run for 1 million generations with four chains. Using the Markov Chain Monte Carlo (MCMC) method, the posterior likelihood of phylogenetic trees was calculated, and consensus trees were created using a 50% majority rule. Consequently, the created trees were visualized and edited using FigTree software v1.4.3. The generated sequences were deposited in GenBank with the accession numbers as shown in Table 1.

## 3. Results

### 3.1. Survey

**Figure 1:**
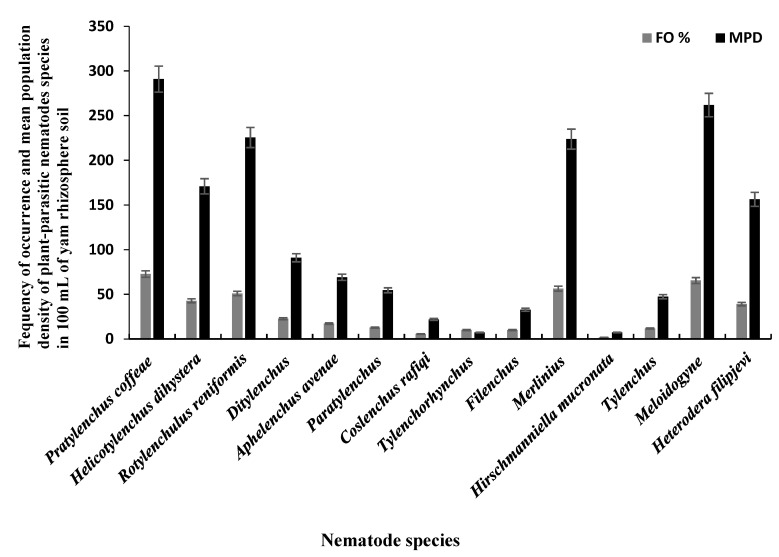
Mean population densities (MPD) and frequencies of occurrence (FO) of plant-parasitic nematodes species recovered from yam rhizosphere in Jiangxi and Shandong provinces of south-east China.

**Figure 2:**
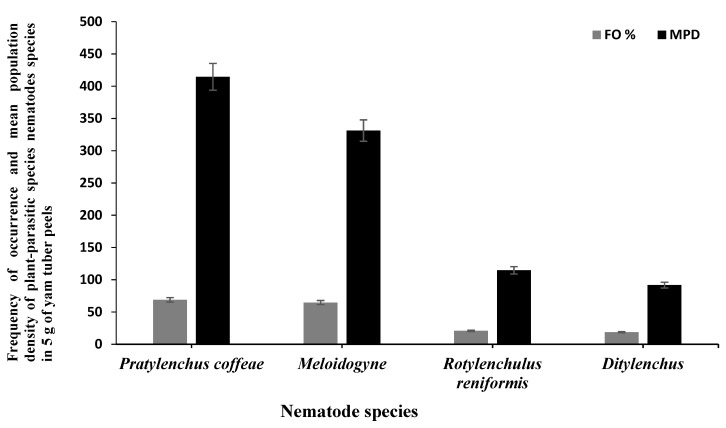
Mean population densities (MPD), and frequencies of occurrence (FO) of plant-parasitic nematodes species recovered from roots/tubers of yam in nine localities of Jiangxi and Shandong provinces across south-east China during 2020/2021.

**Figure 3:**
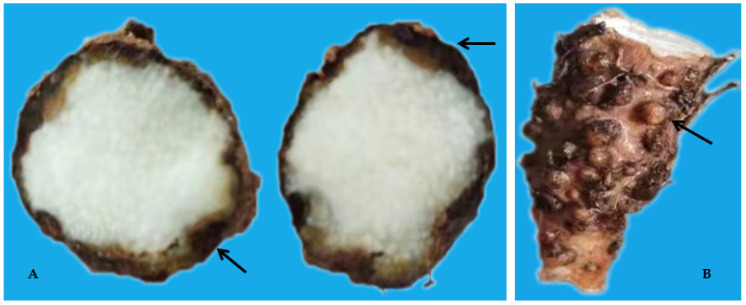
Dry rot (**A**) and Galls (**B**) on yam tubers caused by *Pratylenchus coffeae* and *Meloidogyne* spp.

The 110 bulk soil samples and 48 tubers obtained from nine locations in Jiangxi and Shandong provinces of China yielded 16 species (four species were found in tubers) of PPNs (Table 2 and Table 3). Data pooled for all farming communities showed that *Pratylenchus* (depicted by the species *P*. *coffeae*) had the highest MPD, in both tuber (MPDs of 415 individuals/5 g) and soil (MPD = 291 indiv./100 mL) samples, followed by *Meloidogyne* (*M. incognita*, *M. hispanica*, and *M. ethiopica*), and *Rotylenchulus* (depicted by the species *R*. *reniformis*) (Figure 1, Figure 2 and Figure 3). *Merlinius* was the fourth in soil samples, according to MPDs (Figure 1), and the most commonly recorded genus with ectoparasitic feeding behavior. The least prominent genus in terms of MPDs in tubers was *Ditylenchus* and in soil *Hirschmanniella mucronata* (Figure 1 and Figure 2).

Pooled soil data for all the fields sampled in each province show that *P*. *coffeae* and *Meloidogyne* spp. were the most prevalent PPNs in terms of mean population density (MPD) and frequency of occurrence (FO) (Table 2). The MPDs for *P*. *coffeae* ranged from 276 (Shandong) to 302 (Jiangxi), and this species was present in 69–75% of the fields sampled. *Meloidogyne* was present in 42–100% of the fields with MPDs ranging between 166 (Jiangxi) and 400 (Shandong). *R*. *reniformis* was usually third in dominance with MPDs ranging between 218 (Jiangxi) and 382 (Shandong) and occurred in 55–82% of the fields (Table 2). According to MPDs, the species with the least prevalence was *H*. *mucronata* with MD of 12 in Jiangxi and being present in 3% of the fields while in Shandong, *Tylenchus*, *Filenchus*, and *Tylenchorhynchus* were the least with MPDs of 18 and occurred in 4% of the farms (Table 2). In tubers, the MPDs for *P*. *coffeae* ranged between 145 (Shandong) and 607 (Jiangxi) with individuals identified in fields of each province in up to 35–93% of the samples (Table 3). For *Meloidogyne*, MPDs ranged between 264 (Jiangxi) and 325 (Shandong) individuals in up to 54–80% of the samples. *Ditylenchus*, had low MPDs (ranging between 35 for Shandong and 96 for Jiangxi), with individuals occurring in up to 10–18% of the samples (Table 3).

In this study, we also provide a morphological and molecular characterization of *Merlinius* species while the other four prominent species (*P*. *coffeae*, *Meloidogyne*, *R*. *reniformis*, and *H*. *dihystera*), infesting yam (*Dioscorea* spp.) in Jiangxi and Shandong provinces of China were also verified with appropriate molecular analysis.

### 3.2. Morphological and Molecular Characterizations of Merlinius brevidens

#### 3.2.1. Morphology of *Merlinius brevidens* (Allen, 1955) Siddiqi, 1970 = *Geocenamus brevidens* (Allen, 1955)

**Figure 4:**
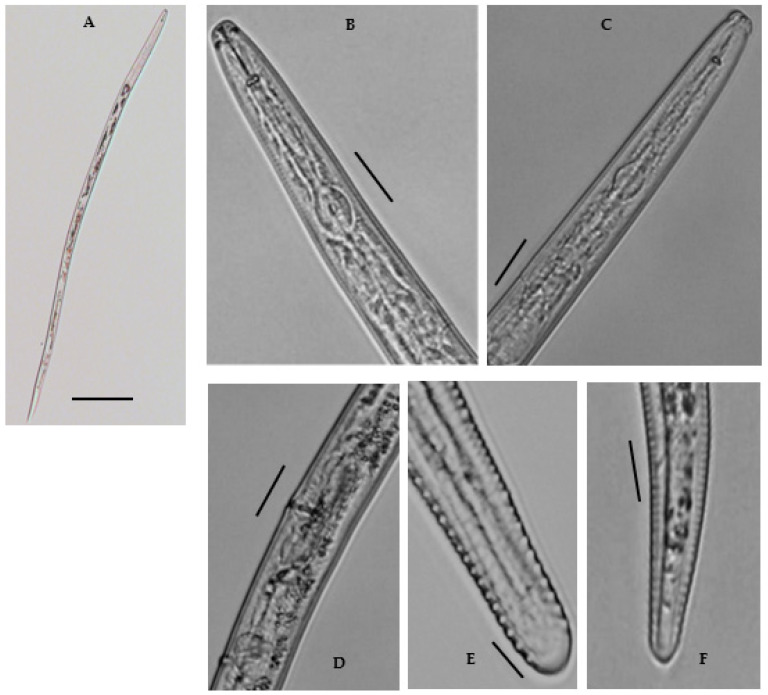
A Photomicrographs of female *Merlinius brevidens*: (**A**) entire female; (**B**,**C**) variation in head region; (**D**) reproductive region; (**E**,**F**) variation in tail region (Scale bars: (**A**) = 200 μm; (**B**–**F**) = 50 μm).

Female: Body shape slightly straight or open C-shaped when heat relaxed with the tapering tail region. The cuticle annulated, lateral field with six incisures. Cephalic region continuous, broadly rounded, 4.6–6.8 μm long and 10.7–12.9 μm wide; with 3–4 indistinct annuli. Stylet robust, with rounded basal sloping knobs, about 2.1–3.8 μm high and 4.4–6.5 μm wide (Figure 4). Median pharyngeal bulb rounded, 4.6–6.0 μm long and 9.0–11.0 μm wide, bearing refractive valve plates, well-developed. Isthmus slender, surrounded by a nerve ring; basal bulb is oval with a small, rounded cardia. Hemizonid prominent, located just anterior to the excretory pore. Excretory pore is anterior to the basal bulb 106.6–114.8 μm long from the end. Vulva transverse, with well-formed epiptygma, protecting the opening of the vulva. Vagina is straight, occupying the ca one-third (20–30%) of the corresponding body width. Didelphic reproductive system; ovaries with oocytes grouped in a single row; distally separated oviducts with rounded spermatheca, filled with sperm; uteri with thin walls as long as the width of the body. Tail subcylindrical with 18–22 annuli, and somewhat set off with bluntly rounded, almost smooth terminus; often acute to bulb-like. The tail hyaline region is conspicuous. Phasmids are distinct, near or slightly posterior to the middle of the tail. No males were found.

Remarks (*n* = 14): The current population analyzed as *M*. *brevidens* from this study morphologically correlates closely with the original description of *M*. *brevidens* by Allen [16], including the morphometric data described by Munawar et al. [27], but differing from both to stylet length (17.4–19.5 μm vs. 14–16 μm, 15–17.5 μm) (Table 4). These diagnostic characters also showed only some morphometric variation to populations described from different geographical regions, particularly in the pharynx length (107.2–124.6 μm vs. Greece isolate, 123–136 μm, and Iranian isolate, 118.5–141 μm) and stylet length (17.4–19.5 μm vs. Greece isolate, 13.0–16.0 μm, Iranian isolate, 16–16.5 μm, Canadian isolates, 15–17.5 μm, and the UK isolate, 14–15.5 μm). The other morphological features, such as lip and tail morphology, overall body habitus, and vulva appearance, were all in line with the original description. Males were mentioned in the initial description by Siddiqi [28], but no males were ever found in later reports. Males were scarce in *M*. *brevidens*, according to Geraert [29], and the studied population was equally devoid of males. Siddiqi [28] also observed tightly closed stylet knobs, an inconspicuous anus, and a cylindrical tail in the population isolated from India. In some of the studied populations, the anus was conspicuous, with rounded stylet knobs and a cylindrical tail with a broadly rounded, somewhat truncated terminus. The morphological variations of the studied population compared with the other isolates can be due to differences in their geographical origin.

The studied population also differs from female *Merlinius microdorus* [30] Siddiqi, [31] by the tail shape (blunt and sub-cylindrical vs. rounded and sub-cylindrical); it is further distinguished by the presence of 18–22 tail annuli with smooth broadly rounded or truncated tail terminus vs. 53–54 tail annuli with a bluntly pointed terminus in *M. microdorus* [18,27]. The studied population can also be distinguished from *Merlinius nanus* [14] Siddiqi [31], by having a robust stylet vs. delicate; blunt and sub-cylindrical tail vs. pointed and conoid tail; tail terminus hemispherical and smooth vs. bluntly pointed and annulated [13,18]. Lateral fields with 6 longitudinal incisures were present vs. 6–10 incisures [13]. Additionally, female tail with 18–22 annuli in present isolates vs. 55–60 in *M. nanus* [13,18]. The differences in morphometric data of females of the present isolate and other described *Merlinius* species are given in Table 4.

#### 3.2.2. Molecular Characterization and Phylogenetic Analysis of *Merlinius brevidens*

The amplification of the D2–D3 fragments of 28S rRNA and ITS-rRNA genes yielded single fragments of approximately 776 and 980–981 bp, respectively. The BLASTN search of the generated D2–D3 sequences of the present strain showed 100% similarities with *M*. *brevidens* Iranian populations (KP313844, KJ585416); 99% similarities with *M*. *brevidens* populations from China (MT856989) and South Africa (MN262457), and 99% similarities with *Merlinius* spp. accessioned KX789700 (Iran) and KY750832 (Mexico); differing from *M*. *brevidens* populations (KP313844, KJ585416, MT856989, and MN262457) by 20–75 bp; and from unidentified *Merlinius* spp. (KX789700, KY750832) by a high genetic distance of between 191 and 476 bp (20–38%). The present strain shows no interspecific differences from the next closest related species (*Pratylenchoides leiocauda* populations; MN539649, MN539650, and MN510993) deposited into GenBank from China, all with 96% identities. The six newly generated ITS sequences showed an intraspecific variation of 1 bp (0.1%), with a relative level of homology to Indian *Merlinius* spp. (MK981336); differing by 106 bp (11%). In addition, genetic distances of between 39 and 43 bp (4%) were also observed when compared with sequences of the closest related population (*Helicotylenchus digonicus*; GQ906352, GQ906353, and GQ906351) from China and Germany.

**Figure 5:**
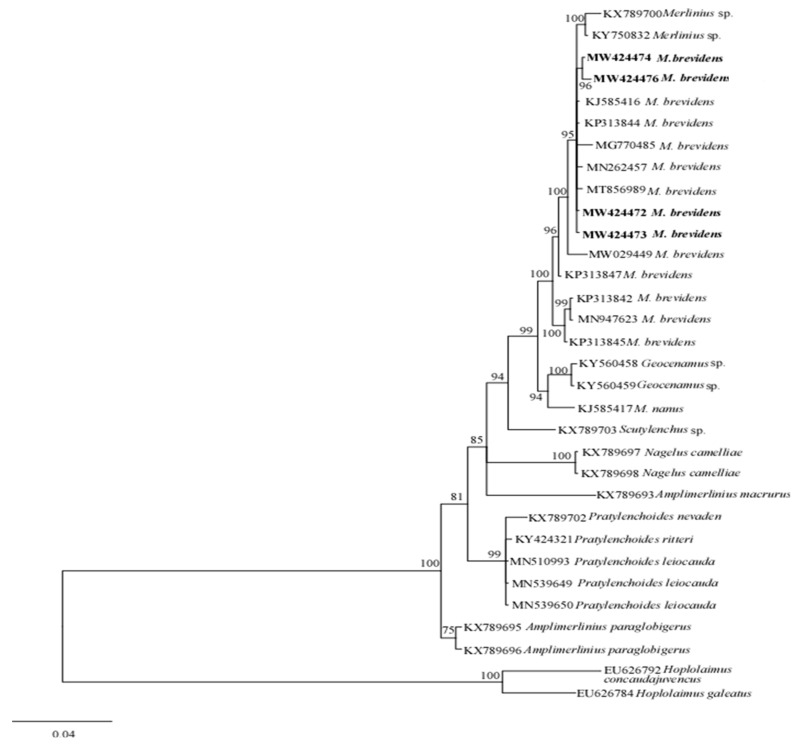
Bayesian tree inferred under the GTR + G + I model from sequences of 28S-rRNA region of the newly sequenced species of Merliniidae indicated in bold and other tylenchids sequences. Posterior probability and bootstrap values exceeding 50% are given on appropriate clades.

**Figure 6:**
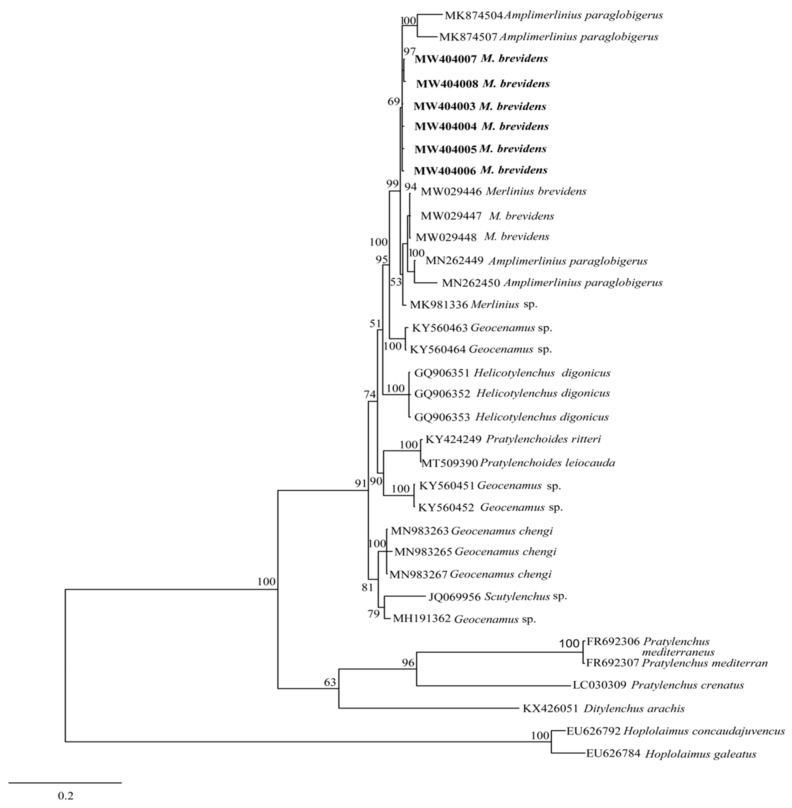
Bayesian tree inferred under the GTR + G + I model from sequences of ITS rRNA region of the newly sequenced species of Merliniidae indicated in bold and other tylenchids sequences. Posterior probability and bootstrap values exceeding 50% are given on appropriate clades.

The phylogenetic relationships between merliniidae and other tylenchids were based on the D2-D3 region of partial sequences from the 28S-rRNA and ITS-rRNA genes, as inferred from Bayesian interference (BI) analysis with general time-reversible (GTR + I + G) substitution model (Figure 5 and Figure 6). The current population formed a well-supported clade with some previously deposited sequences of *M*. *brevidens* (KP313842, KP313844, KP313845, and KJ585416) in NCBI from Iran, with 95–100% bootstrap support values according to the phylogenetic tree inferred from the study of the D2–D3 region of the 28S rRNA gene sequence alignment. They specifically belong to the same clade as other *M*. *brevidens* isolates from Mexico, Greece, Canada, the United States of America, and South Africa, suggesting that they are conspecific. However, a sub-clade of *M*. *brevidens* was formed by a few additional populations from Iran (KP313842, KP313845, KP313847, Alvani et al. [32]; MN947623, unpublished). The authors did not provide morphological or morphometric data for these last populations, implying that they should be re-evaluated using detailed integrative taxonomy. Additionally, *M*. *brevidens* isolates cluster together in the same sister clade as *M*. *nanus* from the United States and appeared as a different, well-supported clade with 100% bootstrap support.

These populations also form a clade with more closely related species of *Amplimerlinius* and *A*. *paraglobigerus* (MK874504 and MK874507) sequences from South Africa, with 69% bootstrap support, and together they formed a sister clade to *M*. *brevidens* from Canada (MW029446-MW029448) and *A*. *paraglobigerus* from S/Africa (MN262449) based on ITS-rRNA sequences. Furthermore, only a few *Merlinius* species have been molecularly identified; the studied *M*. *brevidens* population was also found in the same major clade as *G*. *chengi* and unidentified *Geocenamus* species.

*M*. *brevidens* is a cosmopolitan species that has been reported from numerous countries, according to a literature review [13,27]. However, few or no integrative taxonomic descriptions were included with any of the *M*. *brevidens* sequences that were submitted to GenBank. As a result, determining their genuine identity is challenging.

## 4. Discussion

In this study, we provide the abundance and prevalence of some PPN encountered in major yam growing areas of Jiangxi and Shandong provinces of China. The 17 PPN species recovered in the present study have previously been described in China from a variety of other crops [4,33,34,35,36] and yam in other parts of the world [4,11,33,37]. These five major occurring species (*Pratylenchus coffeae*, *M*. *incognita*, *R*. *reniformis*, *H*. *dihystera*, and *Merlinius brevidens*) on yam based on population density and frequency of occurrence were also verified with appropriate molecular analysis. Additionally, the first report of *M*. *brevidens* from yam in China was confirmed in this study using an integrative approach that looked at morphological, morphometric, and molecular variables.

In China, *P*. *coffeae* tend to be the predominant species on yam, which is consistent with other studies that have found *P*. *coffeae* on yam in China and other Asian countries [4,18,29]. It is among the most economically important nematode threats to yam production worldwide and is most likely the most significant biotic factor threatening yam production in Asia [4,11,33]. It is recognized as one of the common causes of “dry rot” on yam tubers in China, a disorder similar to that caused by *S*. *bradys* [4,11]. These polyphagous species have also been identified as a pest of several crops in this region [4,38,39]. RKN was the second most common species found in yam, followed by *R*. *reniformis*; both of these nematodes have previously been associated with yam in Costa Rica [37], Ghana [4], and Puerto Rico [40]. In China, RKN species were also recorded to be widespread on yam and many other important crop plants in the south to northern regions of the country causing significant yield losses of 70% [41,42] as well as *R*. *reniformis* on tomatoes [36]. *Merlinius* spp. was also among the most frequently encountered species in the rhizosphere soil of both provinces, indicating that it is an exclusively ectoparasitic feeder [13,19]. Ectoparasitic nematodes may play a larger impact on yam than previously thought, given their relatively high frequency of occurrence. However, the economic significance of ectoparasitic nematodes such as *Merlinius* on yam remains unknown [19]. The nematodes in this group are known to cause stunting and chlorotic tillers in cereal crops in the USA [15,27]. In addition, *Merlinius* spp. has also been detected in cultivated cereal and yam fields in Australia and Nigeria, where it has been associated with RLN, RKN, and *S. bradys* [19,27]. The prevalence of *M*. *brevidens* in both agricultural and horticultural fields has been documented in European and Asian countries [27,32], although no significant plant damage has been attributed to this species. We hypothesize that *M*. *brevidens* has a substantially greater impact on crops, based on the reported damage caused by this species in the USA and its association to RLN, RKN, and *S. bradys* in Nigeria. Furthermore, the high prevalence of the five predominant species over others in the present study may be attributed to environmental or host suitability factors [12,43]. Other potentially significant nematode taxa (*Tylenchorhynchus*, *Paratylenchus*, *Filenchus*, *Tylenchus*, and *Coslenchus*) were typically collected only in low densities, and their association with yam is yet to be determined. These taxa were not included on the list of nematodes that are confirmed or suspected of causing yam yield loss [4,11,19]. The occurrence of *Heterodera filipjevi* in yam rhizosphere soil obtained from Shandong and *Hirschanniella mucronata* in Jiangxi were unanticipated, and their association with yam has not previously been established, but they are considered a major pest of wheat (*Triticum* spp.) and rice (*Oryza* spp.) in China [34,44]. However, their absence from tuber tissue suggests that yam tubers do not encourage the growth of *Heterodera filipjevi* and *Hirschmanniella mucronata* and that their presence, in this case, may be attributed to the presence of other plant species in the same region.

*Scutellonema bradys*, one of the most important plant-parasitic yam nematodes in West Africa and the Caribbean, was not found in any of the samples collected from the Jiangxi and Shandong provinces of China. Coyne and Affokpon [4] from China made a similar observation. Despite being found on yam in India and Korea, Coyne and Affokpon [4] claim that “*S*. *bradys* is a pest of yam, surprisingly, not in China”. The nematode is responsible for tuber weight loss during storage, as well as loss of edible portion, tuber quality, and planting material. *S. bradys* is also most notable for the direct damage it causes to tubers [4,19,40]. Tubers infected with *S. bradys* lead to a total loss in synergetic impact with secondary infection [11]. Therefore, infected tubers have little or no marketable value. Additionally, *S*. *bradys* has been recognized for up to 50% of the loss of stored yam tubers, with 100% loss observed in severe situations [4,19]. Similarly, several nematode species (*Pratylenchus brachyurus*, *P*. *zeae*, *P*. *pseudopratensis*, and *P*. *sudanensis*) are also known to damage yam in Africa, with evidence indicating they are rather abundant in the yam rhizosphere and on tubers [12,19].

*Merlinius brevidens* populations were studied using morphological, morphometrical, and molecular data. The study of the D2-D3 segment of 28S rRNA and ITS regions has been used to distinguish *Merlinius* species as well as other reported species [13,32,45]. The present *Merlinius* population showed similar morphology and 100% similarity of D2-D3 sequence with the *M*. *brevidens* from Iran [32]. However, a close analysis of the morphometrics of the original [14] and other reported description by Alvani et al. [32] revealed some conflicting variations from the studied population, with a relatively shorter stylet (14–16 μm, 16–16.5 μm vs. 17.4–19.5 μm) and longer pharynx length (118.5–141 μm vs. 107.2–124.6 μm). Additionally, our morphological and morphometric data of the described *Merlinius* spp. largely agree with a population designated as *M*. *brevidens* in Munawar et al. [27] and Bharti et al. [13]; with accession number: MW029446-MW029448 and MK981336 based on ITS-rRNA sequence similarity and the key morphological characters presented, especially the shorter stylet length (17.4–19.5 μm vs. 15–17.5 μm, 13–14 μm), bluntly rounded lip region, slightly set off from the body contour bearing 3–4 lip annuli, and tail length (41–63 μm). Additionally, in our phylogenetic trees based on the 28S rRNA gene, *M. brevidens* sequences formed two separated sister clades. Our *M*. *brevidens* results are consistent with the original descriptions, as well as other populations characterized using integrative taxonomy [27,32,45]. As a result, more research on the populations that made up the second sub-clade is required. These species are more likely to be related to other *Merlinius* species than to *M*. *brevidens*. We concur with Handoo et al. [46], who noted that stunt nematodes had morphological diversity and that overlapping morphometrical values could lead to misidentification. As a result, the only conclusive solution to this problem will be to sequence the topotype population of *M*. *brevidens*, which may provide insight into the situation. Therefore, we suggest our *M*. *brevidens* as the standard and reference population for this species until topotype specimens are available and molecularly identified, based on integrative taxonomical classification.

## 5. Conclusions

In conclusion, the current research offers an outline of nematode distribution in the major yam-cultivating areas, as well as an update on the present status of nematode pests of yam. It seems that nematodes tend to be a major threat to yam production in both provinces, as significant nematode parasites have been detected in both the tubers and rhizosphere of yams. Consequently, to increase yam productivity, a strong emphasis on reducing these pests on yam crops within the region is needed. Farmers should be informed about the nematode problem and be given options for management, such as rotating resistant crop cultivars with yam, biological control agents, and other approaches to facilitate sustainable production. Accurate diagnosis, on the other hand, is essential for developing and implementing appropriate and effective management strategies. Furthermore, little is known about the relationships or combined effects of the four PPN (*P*. *coffeae*, *Meloidogyne* spp., *R*. *reniformis*, and *Ditylenchus* spp.) species that have been detected on yam tubers. As a result of the interspecific diversity of such species parasitizing yam in China, a wide variety of commercial and wild yam cultivars should be screened to identify germplasm with sufficient resistance or tolerance to them. To determine the virulence and damage thresholds of each species, as well as their combined effects on yam, further investigation is therefore needed.

Additionally, the prevalence and distribution of non-target nematode species in Jiangxi and Shandong provinces of China are currently very rare. We present descriptions of stunt nematode based on an integrative taxonomic approach in this paper. The current research will aid in the updating of *M*. *brevidens* taxonomy records in China. In addition, our light micrographs and DNA-based data will allow us to identify these species quickly. Finally, more research is therefore needed to decide definitively whether or not this stunt nematode should be included in nematode management strategies.

## Figures and Tables

**Table 1:** Plant-parasitic nematodes species on yam collected in south-east China provinces (Jiangxi and Shandong), soil type characterization, and respective GenBank accession number from 28S and ITS regions.

Code	City/County	Province	Soil Type	Species	GenBank Accession Number	
					28S	ITS
360826/THE	Taihe	Jiangxi	Sandy clay	*Pratylenchus coffeae,*	MW082090,	MW042905
				*Rotylenchulus reniformis,*	MW082086	MW042913
				*Helicotylenchus dihystera*	MW082085,	MW042910
				*Merlinius brevidens*	MW424473,	MW404014
				*Meloidogyne incognita*	MW424479,	MW404001
				*Ditylenchus*	-	MW042916
				*Hirschmanniella mucronata,*	>MW424469,	MW042911
				*Tylenchorhynchus annulatus*	MW424463	MW042920
360827/SCN	Suichun	Jiangxi	Sandy clay	*P. coffeae*	MW082091	MW042906
				*R. reniformis,*	MW082086	MW042914
				*M. brevidens*	-	MW404009
				*M. incognita*	-	MW403996
				*Ditylenchus*	-	MW042917
				*H. mucronata*	MW424470	MW042912
360881/JGS	jingganshan	Jiangxi	Clay	*P. coffeae*	-	MW042907
				*R. reniformis*	MW082088	-
				*M. incognita*	-	MW403997
				*M. brevidens*	-	MW404008
				*Aphelenchus avenae*	-	MW042919
				*Tylenchorhynchus zeae*	MW082095	-
360481/RCG	Ruichang	Jiangxi	Sandy loam	*P. coffeae*	-	MW042908
				*R. reniformis*	MW082089,	MW042915
				*M. brevidens*	-	MW404006
				*M. incognita*	MW812359	MW404000
				*T. zeae*	MW082096	-
				*Coslenchus rafiqi*	MW082097	-
360724/SYO	Shanyou	Jiangxi	Sandy loam	*P. coffeae*	-	MW042909
				*R. reniformis*	MW082087	-
				*H. dihystera,*	MW424464	-
				*M. incognita*	-	MW403998
				*M. brevidens*	-	MW404003
				*Filenchus vulgaris*	MW082093	-
360825/YFX	Yongfeng	Jiangxi	Sandy clay	*P. coffeae*	-	MW404011
				*R. reniformis*	MW812367	-
				*H. dihystera*	MW424466	-
				*M. incognita*	MW812358	MW403999
				*F. vulgaris*	MW082092	-
3714/DZS	Dezhou	Shandong	Sandy loam	*P. coffeae*	MW424468	MW404010
				*R. reniformis*	MW812368	-
				*H. dihystera*	MW424465	MW812365
				*Merlinius brevidens*	MW424472	MW404007
				*M. incognita*	MW424478	MW404002
				*Heterodera filipjevi*	MW424477	MW403994
				*Ditylenchus*	-	MW042918
				*M. hispanica*	MW829784	MW812366
				*M. ethiopica*	MW424480	-
3707/WEF	Weifang	Shandong	Sandy loam	*P. coffeae*	-	MW404012
				*R. reniformis*	-	-
				*M. brevidens*	MW424474	-
				*M. incognita*	-	MW404005
				*H. filipjevi*	MW812362	MW403993
				*Ditylenchus*	-	MW404013
				*M. hispanica*	MW829785	-
				*M.ethiopica*	MZ352771	-
3705/DYG	Dongying	Shandong	Sandy	*P. coffeae*	-	-
				*R. reniformis*	-	-
				*M. brevidens*	MW424476	-
				*M. incognita*	MW812357	MW404004
				*H. filipjevi*	MW812361	MW403995
				*M*. *hispanica*	MZ352769	-

**Table 2:** Mean population densities (MPD), and frequencies of occurrence (FO) of plant-parasitic nematodes species isolated from 100 mL of rhizosphere soil sampled from yam plants in nine locations of Jiangxi and Shandong provinces across south-east China during 2020/2021.

			Province		
	Jiangxi (*n* = 65)			Shandong (*n* = 45)	
	MPD	FO (%)		MPD	FO (%)
Nematode species					
*Pratylenchus coffeae*	302	75		276	69
*Meloidogyne* species (*M*. *incognita*, *M*. *ethiopica*, *M*. *hispanica*)	166	42		400	100
*Rotylenchulus reniformis*	218	55		382	82
*Helicotylenchus dihystera*	191	48		142	36
*Merlinius brevidens*	117	29		231	58
*Aphelenchus avenae*	74	18		62	16
*Ditylenchus* spp.	92	23		89	22
*Tylenchorhyncus* (*T*. *annulatus*, *T*. *zeae*)	55	14		18	4
*Filenchus vulgaris*	55	14		18	4
*Tylenchus* spp.	68	17		18	4
*Coslenchus* spp.	37	9			
*Paratylenchus* spp.	68	15		36	9
*Heterodera filipjevi*	-	-		382	96
*Hirschmanniella mucronata*	12	3		-	-

*n* = number of samples, **-** = no nematodes present.

**Table 3:** Mean population densities (MPD) and frequencies of occurrence (FO %) of plant-parasitic nematodes species isolated from 5 g of yam roots/tubers from nine locations of Jiangxi and Shandong provinces of south-east China during 2020/2021.

			Province		
	Jiangxi (*n* = 28)			Shandong (*n* = 20)	
	MPD	FO (%)		MPD	FO (%)
Nematode species					
*Pratylenchus coffeae*	607	93		145	35
*Meloidogyne* species (*M*. *incognita*, *M*. *ethiopica*, *M*. *hispanica*)	264	54		325	80
*Rotylenchulus reniformis*	115	21		110	25
*Ditylenchus* spp.	96	18		35	10

*n* = number of samples.

**Table 4:** Comparative morphometrics of females from populations of *Merlinius brevidens*. All measurements are in micrometers (except n, ratio, and percentage) and in the form: mean ± standard deviation (range).

Character	*Merlinius brevidens*	*M. brevidens*	*M. brevidens*	*M. brevidens*	*M. brevidens*	*M. brevidens*	*M. brevidens*	*M. microdorus*	*M. nanus*
Reference	Present study (China)	Canada: Munawar et al. (2021)	India: Bharti et al. (2020)	Greece: Tzortzakakis et al. (2018)	Iran: Alvani et al. (2017)	UK: Bridge (1971)	USA: Allen (1955)	Belgium: Geraert (1966)	USA: Allen (1955)
N	14	15	10	3	8	10	11	14	8
L	655.3 ± 25.5 (631.3–707.9)	(591–811)	(572–644)	(490–698)	(600–718.5)	(526–676)	(540–690)	(580–700)	(520–640)
a	34.0 ± 2.0 (31.7–39.9)	(25.5–38.5)	(26.1–31.6)	(22.9–30.3)	(23.9–29.0)	(21.5–27)	(23–27)	(24.0–28.5)	(27–31)
b	5.6 ± 0.4 (5.1–6.6)	(4.6–5.9)	(4.2–5.3)	(4.0–5.1)	(4.5–5.2)	(4.5–5.1)	(4.2–5.2)	(4.8–5.9)	(4.5–5.3)
b’	4.9 ± 0.3 (4.5–5.8)	-	(4.7–5.8)	-	-	-	-	-	-
c	13.2 ± 1.9 (10.1–16.3)	(11.7–13.6)	(11.3–15.0)	(12.9–17.0)	(11.3–15.7)	(12–15)	(11–13)	11.0–13.0	(10–12)
c’	3.6 ± 0.7 (2.7–5.4)	(3.8–4.9)	(2.8–4.9)	(2.9–3.2)	(2.4–3.2)	(2.4)	(2.4)	(2.9)	-
MB	53.1 ± 4.6 (45.2–61.1)	(46.2–54.3)	(46–57)	-	(41.1–46.5)	-	-	(41–51)	-
V	59.0 ± 2.0 (54.7–62.4)	(53.8–61.0)	(54–58)	(52.0–58.0)	(54.5–57.0)	(53–58.5)	(52–58)	(55–59)	(52–57)
Lip height	5.3 ± 0.4 (4.6–6.0)	(3.1–4.0)	(3–4)	-	-	-	-	-	-
Lip width	9.9 ± 0.6 (9.0–11.0)	(6.1–7.7)	(6–8)	-	-	-	-	-	-
Stylet length	18.8 ± 0.6 (17.4–19.5)	(15–17.5)	(13–14)	(13.0–16.0)	(16–16.5)	(14–15.5)	(14–16)	(12–14)	(12–15)
Stylet base diameter	15.2 ± 0.5 (14.1–15.9)	-	(12–13)	-	-	-	-	-	-
Anterior to median bulb valve	61.8 ± 3.2 (54.5–65.9)	-	(55–64)	-	-	-	-	-	-
Pharynx length	116.8 ± 6.8 (107.2–124.6)	(120.3–144)	(107–123)	(123–136)	(118.5–141)	-	-	-	-
Anterior to end of pharyngeal glands	134.5 ± 7.0 (123.1–142.0)	-	-	-	-	-	-	-	-
Anterior to excretory pore	112.1 ± 2.6 (106.6–114.8)	-	(84–102)	-	(90.5–110)	-	-	-	-
Maximum body width	19.3 ± 0.7 (17.7–20.5)	(15.5–21.2)	(19–24)	(19–23)	(21.5–30)	-	-	-	-
Anterior end to vulva	386.8 ± 27.3 (345.1–439.1)	-	(313–361)	-	(327–392)	-	-	-	-
Body width at vulva	22.3 ± 1.3 (20.1–24.6)	(16.4–22.3)	(17–24)	-	(21.5–30)	-	-	-	-
Tail length	51 ± 7 (41–63)	(46–59.8)	(41–54)	(38–41)	(42–53)	(46)	-	(34–63)	-
Anal body diameter	14.4 ± 1.4 (11.5–16.3)	(10.3–14.2)	(10–16)	(12–14)	(15.5–19)	(19)	-	(17)	-

## Data Availability

DNA sequence data were deposited in the GenBank database under the accession numbers: MW082085-MW082097, MW424463-MW424480, MW812362-MW812367, MW829784-MW829785, and MZ352769-MZ352771 (28S sequences); MW042905-MW042920, MW403993-MW404013, and MW812366 (ITS sequences).
